# A Wi-Fi/PDR Fusion Localization Method Based on Genetic Algorithm Global Optimization

**DOI:** 10.3390/s25247628

**Published:** 2025-12-16

**Authors:** Linpeng Zhang, Ji Ma, Yanhua Liu, Lian Duan, Yunfei Liang, Yanhe Lu

**Affiliations:** 1 School of Geographical Sciences and Planning, Nanning Normal University, Nanning 530001, China; zhanglinpeng011@email.nnnu.edu.cn (L.Z.); lyf_yfei@nnnu.edu.cn (Y.L.); 2 Key Laboratory of Environment Change and Resources Use in Beibu Gulf, Ministry of Education, Nanning Normal University, Nanning 530001, China; lyh_lucy@nnnu.edu.cn (Y.L.); lianduan@nnnu.edu.cn (L.D.); lyh@email.nnnu.edu.cn (Y.L.); 3 School of Natural Resources and Surveying, Nanning Normal University, Nanning 530001, China

**Keywords:** indoor localization, sensor fusion, Wi-Fi fingerprinting, Pedestrian Dead Reckoning, Genetic Algorithm, global optimization

## Abstract

**Highlights:**

**What are the main findings?**

- A novel Wi-Fi/PDR fusion localization method based on a Genetic Algorithm (GA) is proposed, reformulating the state estimation problem into a global trajectory alignment optimization framework with a robust Huber-based cost.
- The proposed GA-based method achieves the highest geometric consistency and positioning accuracy (mean error 0.878 m, RMSE 0.978 m), outperforming Wi-Fi fingerprinting, PDR, and EKF fusion by 69.6%,31.3%, and 26.4% respectively.

**What is the implication of the main finding?**

- The GA-based global optimization strategy largely removes the dependency on initialization and suppresses non-Gaussian noise, offering a robust and largely initialization-insensitive indoor localization method for multi-sensor fusion.
- This study introduces an optimization-based alternative to traditional recursive filtering, providing a potential foundation for future map-constrained and 3D indoor localization.

**Abstract:**

In indoor environments, fusion localization methods that combine Wi-Fi fingerprinting and Pedestrian Dead Reckoning (PDR) are constrained by the high sensitivity of traditional filters, such as the Extended Kalman Filter (EKF), to initial states and by their susceptibility to nonlinear drift. This study presents a Wi-Fi/PDR fusion localization approach based on global geometric alignment optimized via a Genetic Algorithm (GA). The proposed method models the PDR trajectory as an integrated geometric entity and performs a global search for the optimal two-dimensional similarity transformation that aligns it with discrete Wi-Fi observations, thereby eliminating dependence on precise initial conditions and mitigating multipath noise. Experiments conducted in a real office environment (14 × 9 m, eight dual-band APs) with a double-L trajectory demonstrate that the proposed GA fusion achieves the lowest mean error of 0.878 m (compared to 2.890 m, 1.277 m, and 1.193 m for Wi-Fi, PDR, and EKF fusion, respectively) and an RMSE of 0.978 m. It also attains the best trajectory fidelity (DTW = 0.390 m, improving by 71.0%, 14.7%, and 27.8%) and the smallest maximum deviation (Hausdorff = 1.904 m, 52.4% lower than Wi-Fi). The cumulative error distribution shows that 90% of GA fusion errors are within 1.5 m, outperforming EKF and PDR. Additional experiments that compare the proposed GA optimizer with Levenberg–Marquardt (LM), particle swarm optimization (PSO), and Procrustes alignment, as well as tests with 30% artificial Wi-Fi outliers, further confirm the robustness of the Huber-based cost and the effectiveness of the global optimization framework. These results indicate that the proposed GA-based fusion method achieves high robustness and accuracy in the tested office-scale scenario and demonstrate its potential as a practical multi-sensor fusion approach for indoor localization.

## 1. Introduction

Location-Based Services (LBS) have become a fundamental component of the Internet of Things (IoT) ecosystem, supporting applications such as smart cities, emergency response, and personal navigation. While outdoor positioning largely relies on the Global Navigation Satellite System (GNSS), its signals are severely attenuated indoors due to building obstructions, rendering them unreliable for accurate localization [1,2].

To address this limitation, a variety of indoor positioning technologies have been developed. Among infrastructure-based methods, RSSI-based Wi-Fi fingerprinting [3] is still one of the most widely deployed solutions because it leverages existing wireless networks; however, its accuracy is strongly affected by multipath propagation and dynamic environmental changes, often leading to errors at the 3–5 m level [4]. Bluetooth Low Energy (BLE) localization [5] offers low power consumption but faces trade-offs between deployment cost and coverage [6]. Ultra-Wideband (UWB) technology [7] can reach centimeter-level accuracy under line-of-sight conditions [8], yet its performance degrades in non-line-of-sight (NLOS) scenarios and the equipment cost is relatively high. Radio-Frequency Identification (RFID) systems [9] provide low-cost solutions but typically suffer from limited accuracy and strong susceptibility to interference [10]. As a complementary approach, Pedestrian Dead Reckoning (PDR) utilizes the inertial measurement unit (IMU) embedded in mobile devices to continuously estimate pedestrian motion [11]. PDR can deliver smooth local trajectories, but its error accumulates rapidly over time and it lacks absolute global coordinates [12].

Despite the rapid progress of ranging and fingerprinting techniques, such as Wi-Fi RTT, channel state information (CSI), ultra-wideband, and vision-based methods, a large portion of already deployed indoor positioning systems still rely on conventional RSSI-based Wi-Fi fingerprinting. In many commercial buildings, access points provide only received signal strength indicators, whereas RTT/CSI measurements require hardware or firmware upgrades and ultra-wideband anchors are rarely available. Consequently, recent research does not abandon RSSI fingerprinting, but instead attempts to mitigate its well-known limitations from different angles. On the modelling side [13], employs a neural-network-based localization method to directly learn a nonlinear mapping from RSSI fingerprints to positions [14], uses a semi-supervised GAN to synthesize labeled fingerprints and thus improve performance in multi-floor buildings, and [15] applies functional discriminant analysis for adaptive long-term Wi-Fi localization and radio-map maintenance. On the system and data-collection side [16], shows that carefully handling last-seen timestamps and stale RSSI readings is critical for robust localization [17], proposes a low-overhead collaborative fingerprinting scheme that explicitly accounts for device heterogeneity between users and access points, and [18] introduces a sequence-to-sequence RSSI framework that estimates pedestrian location sequences from unconstrained RSSI trajectories in general environments. Taken together, these studies demonstrate that RSSI-based fingerprinting remains an active research topic and confirm that, in practice, deployed systems often still have to work with sparse and noisy RSSI-only position fixes whose typical errors remain on the order of several meters.

In this work, we intentionally adopt this challenging yet realistic setting: Wi-Fi RSSI fingerprinting is used as a representative source of such sparse and noisy global observations. Our goal is not to advance Wi-Fi fingerprinting itself, but to develop a trajectory-level fusion framework that can turn these imperfect position fixes into a globally consistent pedestrian track when combined with PDR.

These complementary characteristics naturally motivate multi-sensor fusion to improve both global consistency and local precision [19]. Filtering-based fusion methods, such as the Extended Kalman Filter (EKF) and its variants [20,21], are among the most widely used solutions. In these recursive frameworks, PDR is used for state prediction and Wi-Fi measurements provide observation updates. With careful tuning, EKF-based fusion can achieve sub-meter accuracy in controlled scenarios. However, its performance is highly dependent on the accuracy of the initial state, which is often difficult to obtain in practice. Inaccurate initialization or severe non-Gaussian Wi-Fi errors may lead to slow convergence or even divergence, severely degrading localization accuracy and motivating the search for initialization-free alternatives.

Batch trajectory optimization has been proposed as a promising alternative [22,23]. Instead of updating the state recursively, this class of methods processes all measurements within a time window as a single batch, estimating the entire trajectory by solving a global optimization problem and thereby avoiding explicit initialization. Nevertheless, existing global optimization approaches still face two major challenges. First, the commonly used Euclidean-distance cost function is highly sensitive to outliers in Wi-Fi positioning results. Second, most methods rely on gradient-based local optimizers, which are at risk of becoming trapped in local minima when the objective function is highly non-convex.

To overcome these issues, this paper proposes a Wi-Fi/PDR fusion algorithm based on global geometric alignment optimized via a Genetic Algorithm (GA). The localization problem is reformulated as estimating a two-dimensional similarity transformation that aligns the locally consistent PDR trajectory with the globally referenced but noisy Wi-Fi trajectory. By treating the PDR trajectory as an integrated geometric entity, the GA searches for the optimal rotation, scale and translation parameters, achieving fusion localization without explicit initialization. In addition, a Huber-based robust cost is designed to suppress Wi-Fi outliers, and we systematically compare GA with LM, PSO and closed-form Procrustes alignment to clarify the role of different optimizers.

The main contributions of this paper are as follows:

(1) We propose a fusion method based on global geometric alignment that combines a locally consistent PDR trajectory with sparse, noisy absolute position measurements without requiring an initial state. In this paper, the absolute positions are provided by RSSI-based Wi-Fi fingerprints, but the formulation is essentially generic and can likewise be applied to other data sources such as Wi-Fi RTT, UWB, BLE, and geomagnetic fingerprints.
(2) We design a robust cost function that incorporates the Huber loss together with regularization terms on scale and rotation. This cost function significantly reduces the negative impact of Wi-Fi positioning outliers on the overall trajectory estimation and further enhances the robustness of the algorithm.
(3) We adopt a GA as a gradient-free global optimizer for the alignment problem and systematically compare it with LM, PSO and Procrustes closed-form alignment. Experiments in a real office environment demonstrate that the proposed GA-based fusion outperforms Wi-Fi fingerprinting, PDR and EKF fusion in both accuracy and robustness, while maintaining stable performance under severe Wi-Fi outliers.

## 2. Related Work

### 2.1. Advancements and Challenges in Filtering Based Positioning

To enhance filtering performance, existing research has introduced improvements from multiple perspectives. A first direction is to adapt the filter parameters to environmental changes. For instance, Li et al. [20] designed an adaptive EKF that adjusts process and measurement noise through a dynamic weighting mechanism; their AEKF variant reduced the root-mean-square error (RMSE) to 0.763 m and 0.884 m in two typical indoor layouts. In [24], PDR was combined with time-difference-of-arrival (TDOA) estimation using adaptive distance weighting to suppress cumulative errors, achieving a mean localization error of 0.1–0.3 m in scenario tests. To address the approximation error of EKF in highly nonlinear settings, an Unscented Kalman Filter (UKF) was employed in [25] to obtain higher state-estimation accuracy. In addition, fusion methods based on Particle Filters (PF) have been extensively studied to cope with non-Gaussian noise [26]. A visual navigation system in [27] further demonstrated that filtering algorithms can flexibly fuse information from diverse sensors.

Another line of research focuses on improving the overall robustness and reliability of filtering algorithms by introducing external models or learning components. In [28], an LSTM network was used to learn and predict the PDR trajectory to suppress cumulative errors, while a BP neural network corrected the EKF output, resulting in a mean localization error of 1.18 m. In [29], PDR, geomagnetic data and Wi-Fi were fused via a Robust Extended Kalman Filter (REKF) to counteract performance degradation caused by significant anomalies in observation data, achieving a mean error of 0.69 m. To tackle the high error rate of geomagnetic matching [30], designed a multilevel quality-control mechanism, which reduced the root mean square (RMS) position error by 13.3–55.2% compared with the baseline method.

Although these studies have significantly advanced filtering-based indoor localization, most of them still operate within the recursive filtering framework and therefore do not circumvent a core issue: the strong dependency on the system’s initial state. To mitigate initial misalignment in low-cost strapdown inertial navigation systems (SINS), for example, [31] proposed a strong-tracking UKF whose parameters were tuned using a hybrid PSO-BAS optimization scheme. Such complex designs further highlight that the initial-value problem is an intrinsic limitation of traditional filters, motivating the exploration of batch optimization-based alternatives.

### 2.2. Global Trajectory Optimization and Constraint-Based Fusion

To overcome the initial-value dependency of filtering methods, another important line of work adopts optimization-based formulations. These methods batch-process all measurements over a period and estimate a globally consistent trajectory by minimizing a suitable cost function. A typical strategy is to align the locally precise PDR trajectory with the globally referenced but noisy Wi-Fi trajectory through a geometric transformation, while treating each PDR step displacement as a kinematic constraint between nodes.

Graph-based methods provide a flexible framework for incorporating additional constraints. In a visible-light fingerprinting system proposed by [32], external observations from Wi-Fi, BLE and visible light were added to the factor graph as global position priors, achieving a localization accuracy of around 0.9 m. In [33], a globally optimal trajectory estimate was obtained by optimizing the entire factor graph using nonlinear least squares, with XYZ position errors all below 0.1 m in challenging scenarios. Pedestrian turning behavior was incorporated as an extra constraint in [34], and ref. [35] explicitly aligned and corrected trajectories at the track level, improving position accuracy by 46.3% in a complex campus environment. To handle point mismatches caused by different sampling rates when matching two trajectories [36], introduced an asymmetric semi-Cauchy noise model in graph optimization to deal with UWB NLOS errors, achieving time-consistent localization with a mean absolute accuracy of about 0.3 m. The work in [37] employed the Huber loss to enhance robustness against RSSI fluctuations in Wi-Fi-based localization. These studies collectively underscore the importance of robust cost functions and constraint modelling in optimization-based fusion.

To avoid local minima in complex objective landscapes, metaheuristic algorithms such as Ant Colony Optimization (ACO) and GA have also been explored in indoor localization. The pioneering work in [38] applied ACO to map matching. In [39], a new overlapping multi-state model was proposed and GA was used to optimize the signal propagation model in a real indoor environment, achieving a mean localization error of 1.92 m. A log-distance signal propagation model optimized by GA and updated online in real time was reported in [40], yielding a mean localization error of 2.52 m. These results validate the feasibility of metaheuristic algorithms, especially GA, for solving complex optimization problems in indoor localization. However, existing applications mainly focus on optimizing auxiliary parameters such as signal propagation models, rather than directly estimating user state and performing trajectory alignment. This observation provides an important motivation for our work, where GA is applied to the global trajectory optimization problem itself.

## 3. Wi-Fi/PDR Fusion Based on Global Trajectory Optimization

**Figure 1:**
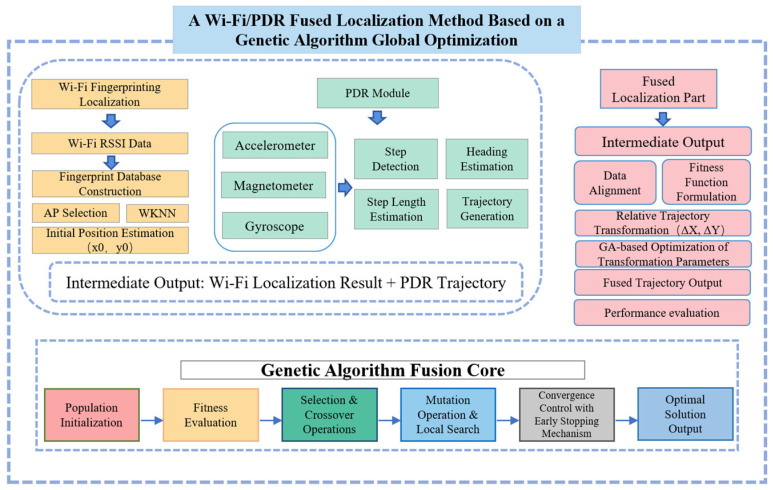
Overall System Framework.

As illustrated in Figure 1, to overcome the limitations of traditional fusion localization algorithms—in particular EKF based methods that are highly sensitive to the initialization of the system state—this paper proposes a Wi-Fi/PDR fusion localization algorithm based on a genetic-algorithm-driven global trajectory optimization. The key idea is to reformulate heterogeneous data fusion as a global optimization problem. By estimating an optimal two-dimensional similarity transformation in the GA fusion core, the method tightly aligns the locally high-precision PDR trajectory with the globally referenced Wi-Fi fingerprinting trajectory, thereby improving both the overall accuracy and the robustness of the final fused localization result.

### 3.1. Data Acquisition for Fusion Localization

#### 3.1.1. Global Trajectory Acquisition via Wi-Fi Fingerprinting

**Figure 2:**
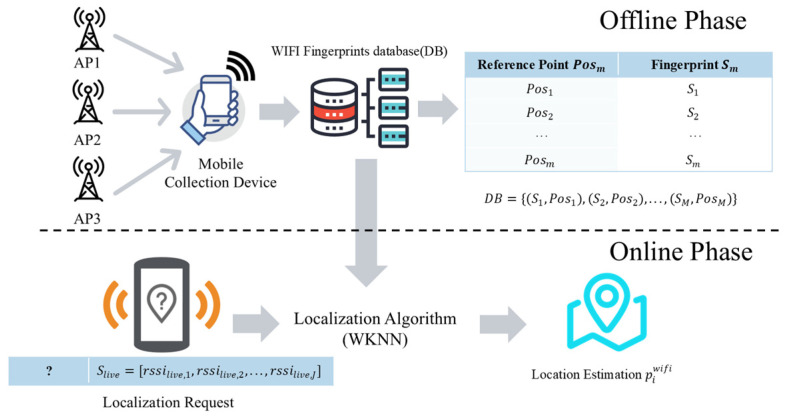
Illustration of the Wi-Fi Fingerprinting Process.

The global reference coordinate system is obtained through the Wi-Fi fingerprinting technique. As shown in Figure 2, this technique is divided into an offline phase and an online phase. In the offline phase, we collect Received Signal Strength Indicator (RSSI) vectors at Reference Points (RPs) with known coordinates in advance to construct a fingerprint database. In the online phase, the user’s position is estimated by matching a real-time RSSI vector against this database.

The main task of the offline phase is to build a fingerprint map by collecting signal-strength values at various coordinate points within the test area. First, M RPs with known coordinates are selected within the localization area. At each RP, the RSSI values from all J detectable Access Points (APs) are collected and organized into a J-dimensional RSSI vector, which serves as the fingerprint $S_{m}$ for that location:

(1) $$S_{m}=[rssi_{m,1},rssi_{m,2},\ldots ,rssi_{m,J}]$$

All RPs’ positions and their corresponding fingerprints together form the fingerprint database DB, which is fundamental for the subsequent online localization:

(2) $$DB=\{(S_{1},Pos_{1}),(S_{2},Pos_{2}),\ldots ,(S_{M},Pos_{M})\}$$

where DB is the constructed fingerprint database, $S_{m}$ is the sequence of signal strengths from J different APs at the m-th fingerprint coordinate point, and $Pos_{m}$ is the true coordinate of that fingerprinting point, for m=1,…,M.

During the online localization phase, the user collects a real time RSSI vector $S_{live}$ at an unknown position that needs to be located:

(3) $$S_{live}=[rssi_{live,1},rssi_{live,2},\ldots ,rssi_{live,J}]$$

In the matching process, we employ the Weighted K Nearest Neighbors (WKNN) algorithm. The WKNN algorithm identifies the K nearest neighboring fingerprints in the signal space based on Euclidean distance. The final position is then calculated by taking a weighted average of the coordinates of these K neighbors, where the weights are the inverse of their respective distances.

The output of this stage is a set of trajectory points $P_{wifi}$ with global coordinates, but which contains noise and outliers:

(4) $$P_{wifi}=\{p_{1}^{wifi},p_{2}^{wifi},\ldots ,p_{N}^{wifi}\}$$

The trajectory obtained from Wi-Fi fingerprinting is susceptible to signal fluctuations and thus has low accuracy. However, it serves as a crucial global coordinate constraint in the subsequent optimization process.

#### 3.1.2. Local Trajectory Estimation via PDR

**Figure 3:**
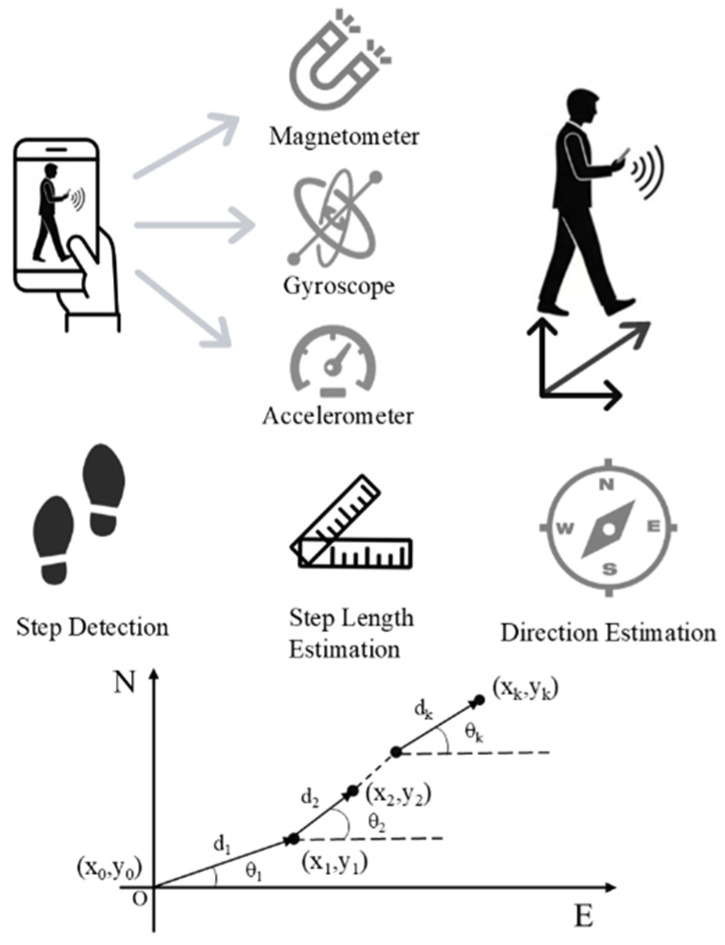
Architecture of the PDR Algorithm.

To capture high-precision local motion dynamics, this work employs the PDR technique. It uses the Inertial Measurement Unit (IMU) embedded in mobile devices such as smartphones to estimate a pedestrian’s relative movement trajectory. The PDR process comprises three core modules: step detection, step-length estimation, and heading estimation. Its overall architecture is illustrated in Figure 3.

Step detection forms the foundation of the PDR algorithm and is performed by analyzing the magnitude of the accelerometer signal. The first step of the algorithm is to calculate the magnitude $a_{mag}(t)$ of the triaxial acceleration $\left(a_{x},a_{y},a_{z}\right)$:

(5) $$a_{mag}(t)=\sqrt{a_{x}(t{)}^{2}+a_{y}(t{)}^{2}+a_{z}(t{)}^{2}}$$

To accurately extract gait-related features, the accelerometer magnitude signal $a_{mag}(t)$ is smoothed using a moving-average filter with a window length of L=5 samples. In our experiments, the IMU sampling rate is set to 100 Hz, and the sampling interval and the corresponding duration of the moving-average window are given by (6) and (7), respectively:

(6) $$\Delta t=\frac{1}{f_{s}}$$

(7) $$T=L\cdot \Delta t=\frac{L}{f_{s}}$$

where $f_{s}$ denotes the IMU sampling frequency, Δt is the sampling interval, and T is the duration of the smoothing window.

By setting a suitable peak threshold factor for the acceleration, each valid walking step can be effectively detected. A time point is identified as the moment a step occurs if it satisfies the following conditions:

(8) $$\left\{\begin{array}{l} {\bar{a}}_{max}(t_{k})>a_{th},\\ {\bar{a}}_{max}(t_{k})>{\bar{a}}_{max}(t_{k-1}),\\ {\bar{a}}_{max}(t_{k})>{\bar{a}}_{max}(t_{k+1}).\;\;\;\; \end{array}\right.$$

After detecting the k-th step, its step length $L_{k}$ needs to be estimated. In this paper, we adopt a widely used non-linear empirical model proposed in the Analog Devices application note AN-602 [41] for step-length estimation:

(9) $$L_{k}=C\cdot \sqrt[{4}]{a_{max,k}-a_{min,k}}$$

where $a_{max,k}$ and $a_{min,k}$ are the maximum and minimum values of the vertical acceleration within the k-th gait cycle, respectively, and C is a personalized calibration coefficient.

To ensure stable and accurate heading, we employed the Madgwick Attitude and Heading Reference System filter. This filter operates in a quaternion framework and effectively suppresses the cumulative drift of the gyroscope by fusing accelerometer and magnetometer data, thereby avoiding the gimbal lock problem. The attitude update process can be summarized as:

(10) $${\dot{q}}_{est,t}={\dot{q}}_{gyro,t}-\beta \nabla f$$

where ${\dot{q}}_{est,t}$ is the final estimated rate of change of the quaternion, ${\dot{q}}_{gyro,t}$ is the rate of change derived from the gyroscope’s angular velocity, ∇f is the gradient representing the measurement error from the accelerometer and magnetometer, and β is the filter gain that determines the correction speed. The yaw angle, which is the required heading angle, is extracted from the solved attitude quaternion after finding the direction that minimizes the error function f using a gradient descent method.

#### 3.1.3. Construction of Wi-Fi–PDR Correspondences

By setting the initial position P_0_ to the coordinate origin (0,0) and iteratively accumulating the displacement vector of each subsequent step, a complete relative motion trajectory starting from (0,0) is generated. This set of trajectory points, denoted as $P_{pdr}$, possesses a high fidelity local geometric shape but contains no global coordinate information and is subject to cumulative drift.

Let $t_{i}$ denote the time stamp of the i-th Wi-Fi scan. The PDR algorithm runs at 50–100 Hz and outputs a high-rate pose sequence $p^{pdr}(t)$. We first reconstruct a continuous PDR path and then resample it at the Wi-Fi time stamps by linear interpolation:

(11) $$P_{i}^{pdr}=p^{pdr}(t_{i})$$

This yields two trajectories $\{p_{i}^{wifi}{\}}_{i=1}^{N}$ and $\{P_{i}^{pdr}{\}}_{i=1}^{N}$ defined on the same set of time instants and along the same physical loop. Therefore, the subsequent alignment operates on point pairs corresponding to the same user position on the loop, and the residuals reflect only the localization errors of the Wi-Fi and PDR subsystems.

### 3.2. Fusion Localization Based on Genetic Algorithm for Trajectory Optimization

After obtaining the globally sparse Wi-Fi trajectory $P_{wifi}$ and the locally precise PDR trajectory $P_{Pdr}$, we employ a global optimization process to find an optimal set of transformation parameters that achieves the best alignment between the two trajectories.

#### 3.2.1. Geometric Transformation Model for Trajectory Alignment

This paper transforms the trajectory alignment task into a problem of solving for an optimal two dimensional similarity transformation. The objective is to find the optimal parameter $X=[\alpha ,\theta ,t_{x},t_{y}{]}^{T}$ set for this transformation. This transformation maps any point $p_{i}^{pdr}$ on the PDR trajectory to the global coordinate system ${\hat{p}}_{i}^{pdr}$, and the similarity transformation is defined as follows:

(12) $${\hat{p}}_{i}^{pdr}=R(\theta )\cdot (\alpha \cdot p_{i}^{pdr})+T=\left[\begin{array}{cc} cos\theta & -sin\theta \\ sin\theta & cos\theta \end{array}\right]\left[\begin{array}{c} \alpha \cdot x_{i}\\ \alpha \cdot y_{i} \end{array}\right]+\left[\begin{array}{c} t_{x}\\ t_{y} \end{array}\right]$$

The transformation is determined by the following three core parameters: α is the scale factor, which compensates for systematic errors in the PDR step length estimation. θ is the rotation angle, which corrects the overall heading deviation of the PDR trajectory via a standard 2D rotation matrix R(θ). $[t_{x},t_{y}{]}^{T}$ is the translation vector, which aligns the origin of the PDR trajectory to the actual starting position in the global coordinate system.

#### 3.2.2. Definition of the Optimization Objective

The optimization objective is to minimize the overall error between the transformed PDR trajectory and the Wi-Fi trajectory. The cost function J(X) is defined in (13).

(13) $$J(X)=\sum_{i=1}^{N}\;L_{\delta }(r_{i})+\lambda_{1}|\alpha -1|+\lambda_{2}|\theta |$$

In this paper, J(X) denotes the total alignment cost associated with a candidate parameter vector $X=[\alpha ,\theta ,t_{x},t_{y}{]}^{T}$. The vector X contains the scale factor α, the rotation angle θ, and the 2D translation $\left(t_{x},t_{y}\right)$ of the similarity transformation that maps the original PDR trajectory into the global Wi-Fi coordinate frame. The optimization problem is to find the parameter set X that minimizes this cost.

(14) $$r_{i}={∥{\hat{p}}_{i}^{pdr}-p_{i}^{wifi}∥}_{2}$$

Here, $r_{i}$ is the residual of the i-th matched point pair, defined as the Euclidean distance between the transformed PDR position ${\hat{p}}_{i}^{pdr}$ and the Wi-Fi reference position $p_{i}^{wifi}$. The notation $∥\cdot {∥}_{2}$ denotes the L2 norm in the horizontal plane, so $r_{i}$ has the unit of meters. In total, N point pairs are used to evaluate the quality of a given transformation X.

(15) $$L_{\delta }(r_{i})=\left\{\begin{array}{ll} \frac{1}{2}r_{i}^{2}, & |r_{i}|\leq \delta ,\\ \delta \left(|r_{i}|-\frac{1}{2}\delta \right), & |r_{i}|>\delta , \end{array}\right.$$

The data term $L_{\delta }(r_{i})$ adopts the Huber loss. For small residuals $\left(|r_{i}|\leq \delta \right)$, it takes the quadratic form $\frac{1}{2}r_{i}^{2}$, which is equivalent to a standard L2 loss and encourages precise alignment when the Wi-Fi measurements are reliable; for large residuals $\left(|r_{i}|>\delta \right)$, it switches to the linear form $\delta (|r_{i}|-\frac{1}{2}\delta )$, so the penalty grows only linearly with $\left|r_{i}\right|$. The threshold δ controls the transition between these two regimes. This piecewise design reduces the impact of occasional large Wi-Fi errors and makes the alignment more robust to outliers. In our implementation, the threshold δ is set adaptively to the median of the current alignment errors. For our data, δ typically lies in the range of 0.5–0.8 m, which is comparable to the characteristic Wi-Fi positioning error and yields stable performance across the test sequences.

The second and third terms in J(X) are regularization terms. The coefficient $\lambda_{1}$ penalizes deviations of the scale factor α from one, reflecting the prior that the average step length of the PDR trajectory should be consistent with the spatial scale of the Wi-Fi coordinate system. Similarly, $\lambda_{2}$ penalizes large rotation angles θ, discouraging unrealistic rotations of the PDR track. These regularizations prevent the optimizer from over-fitting the noisy Wi-Fi measurements by using extreme similarity transforms.

(16) $$X^{*}=arg\;\underset{X}{min}J(X)$$

Therefore, the final goal of the proposed optimization is to estimate the optimal parameter vector $X^{*}$ that minimizes the above cost function $J\left(X\right)$, providing a good trade-off between alignment accuracy, robustness to outliers, and physically reasonable transformation parameters.

#### 3.2.3. Solving the Optimization with the Genetic Algorithm

**Figure 4:**
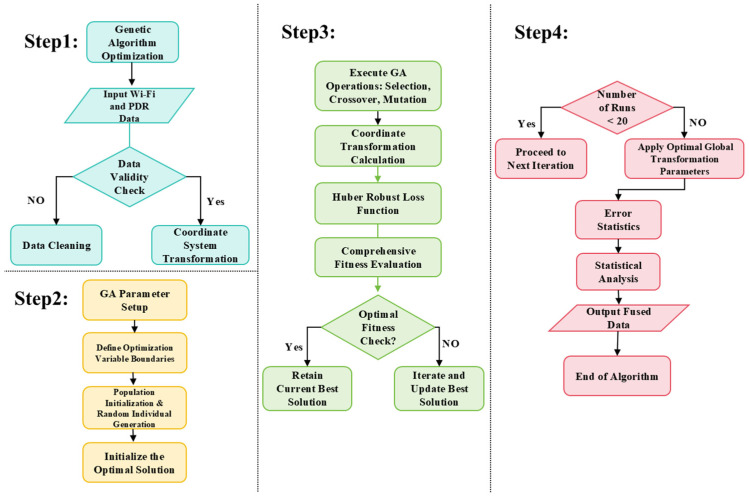
Flowchart of the GA.

Since the aforementioned cost function is complex and nonconvex, traditional gradient based optimization methods are highly susceptible to getting trapped in local optima. Therefore, we chose to employ a GA to solve it. The GA mimics the “survival of the fittest” principle from biological evolution, searching for the global optimum through iterative operations of selection, crossover, and mutation. Its detailed execution process is illustrated in Figure 4.

As illustrated in Figure 4, the execution process of the GA can be broken down into the following four steps:

Step 1: Data Preprocessing and Transformation

The inputs of the algorithm are the raw Wi-Fi trajectory and the raw PDR trajectory.

First, a data validity check is performed on both data streams. For Wi-Fi, we discard scans with missing timestamps, fewer than a predefined number of access points, or obviously inconsistent RSSI values. For PDR, we remove steps with impossible step lengths or heading changes (e.g., extremely small or extremely large values caused by sensor saturation). After filtering, the Wi-Fi and PDR sequences are synchronized in time and re-indexed so that each Wi-Fi position sample has a unique counterpart in the PDR trajectory. This yields N matched point pairs $\{p_{i}^{wifi},p_{i}^{pdr}{\}}_{i=1}^{N}$, which are then transformed into the same 2D global coordinate frame. This preprocessing stage provides clean and consistent input for the subsequent optimization.

Step 2: GA Parameter Setting and Population Initialization

In this step, we define the optimization variables and configure the GA. The optimization variables are collected in the vector $X=[\alpha ,\theta ,t_{x},t_{y}{]}^{T}$, where α is the scale factor, θ is the rotation angle, and $\left(t_{x},t_{y}\right)$ is the 2D translation. The detailed GA configuration used in all experiments is summarized in Table 1, and an initial population of candidate solutions is generated uniformly at random within these bounds. For each individual, the cost function J(X) in Section 3.2.2 is evaluated once to obtain its initial fitness.

Step 3: Core Iteration and Fitness Evaluation

In each generation, a new population is produced by selection, crossover and mutation. For every individual X, the corresponding similarity transform is applied to the PDR trajectory to obtain the transformed positions ${\hat{p}}_{i}^{pdr}$. The residuals are computed for all matched pairs, and the total cost J(X) is evaluated using the Huber loss with threshold δ plus the regularization terms on α and θ. A smaller cost means a higher fitness.

An elitist strategy is used: the best individual in each generation is copied to the next one, and the global best solution is updated whenever a better individual appears. To further reduce the risk of local minima, the GA is run several times with different random seeds, and the best solution over all runs is retained.

Step 4: Convergence check and result generation

The algorithm stops when the maximum number of generations is reached or when the improvement of the best cost falls below a preset threshold. At termination, the global best parameter vector $X^{*}$ with the minimum value of J(X) is returned. The associated similarity transformation is then applied to the entire PDR trajectory to obtain the aligned path, which is used in the subsequent fusion and performance evaluation.

## 4. Experimental Design

To systematically validate and evaluate the performance of the proposed GA based fusion localization method, this chapter will detail the experimental setup, including the test environment, hardware system, dataset construction process, and performance evaluation scheme.

### 4.1. Experimental Environment Setup

**Figure 5:**
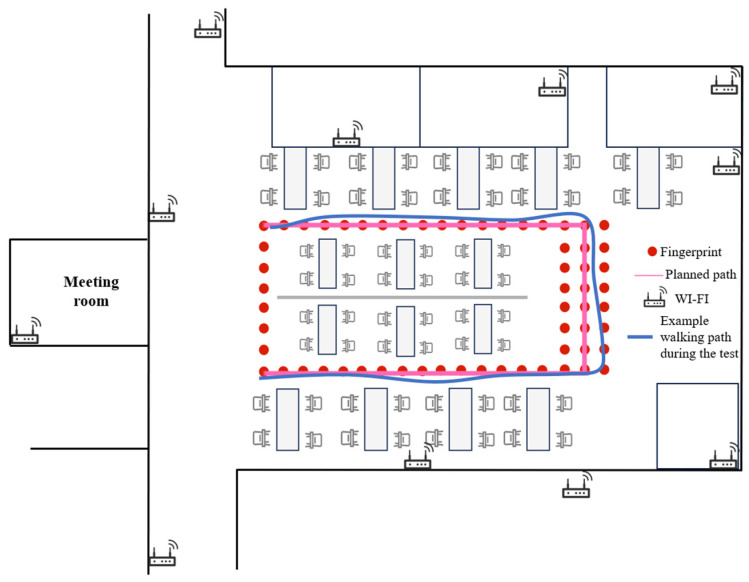
Schematic of the Experimental Environment.

This experiment was conducted in an indoor office environment with an area of approximately 14 m × 9 m. As shown in Figure 5, the scene consists of an open-plan desk area surrounded by a corridor and a small meeting room, with numerous computers and electronic devices creating a complex electromagnetic environment with noticeable multipath effects. The office floor is already covered by the building’s Wi-Fi infrastructure from multiple operators. These existing access points were kept unchanged during the experiments and provide realistic background radio coverage that is typical for many office deployments.

To establish a controlled and reproducible experimental testbed on top of this public network, we additionally deployed eight off-the-shelf dual-band Wi-Fi routers (4 Xiaomi AX3000T, Xiaomi Corporation, Beijing, China, and 4 TP-Link AX3000, TP-Link, Nanshan, Shenzhen, China) at convenient power outlets along the walls, as schematically illustrated in Figure 5. All additionally installed APs were configured with the same transmit power, channel, and bandwidth so that they behave as generic infrastructure rather than being tuned to a specific trajectory. The main wireless parameters of these experimental APs are summarized in Table 2.

During offline processing (Section 4.2), we did not limit the analysis to these experimental APs. Instead, we considered all visible BSSIDs from both the existing building infrastructure and the deployed routers. For each BSSID, we counted the number of reference points (RPs) at which it was detected and computed its mean RSSI level. Based on this coverage-and-stability analysis, we then selected eight BSSIDs that were visible along almost the entire loop and exhibited relatively strong and stable RSSI values as effective anchors for fingerprinting. This data-driven selection ensures that the fingerprint representation is determined by signal coverage and stability, not by manual tuning for the test path or by a fixed assumption on the number of APs.

### 4.2. Experimental Data Collection

In the experimental area, we used the 1 m × 1 m floor grid as a reference and manually selected 60 reference points (RPs) along a closed loop surrounding the central desk area (red dots in Figure 5). These RPs lie on the corridor centerline and define a planned reference path for the fingerprint survey rather than the exact footsteps of the user, as illustrated in Figure 5 by the red dots and pink dashed line. At each RP, the tablet collected Wi-Fi scans at 1 Hz for 2 min. During this site survey, dozens of BSSIDs from both the existing building Wi-Fi infrastructure and the additional routers were observable at different locations. To obtain a compact yet informative fingerprint representation, we evaluated each BSSID by counting the number of RPs at which it was detected and by computing its mean RSSI level. We then selected eight BSSIDs that were visible at most RPs and exhibited relatively strong and stable RSSI values, and used them as effective anchors for positioning. The fingerprint vector at each RP therefore contains only the RSSI values of these eight APs, while very weak or highly intermittent BSSIDs are discarded as they mainly introduce noise.

To evaluate the performance of the proposed algorithm, we instructed a test subject to walk one lap along the corridor around the desk area, roughly following the planned reference path but without constraining the footsteps to the discrete RPs. The blue curve in Figure 5 illustrates an example walking path during the test, which fluctuates slightly around the ideal centerline, as is typical for human walking. The subject walked at approximately 1 step/s and held the tablet in a natural handheld posture, producing 73 consecutive steps. During the walk, the IMU sensor data used for PDR and the Wi-Fi RSSI data were recorded synchronously. Because of the latency and caching of the operating system’s Wi-Fi scanning mechanism, several consecutive scans may sometimes return identical BSSID–RSSI vectors. Therefore, before algorithmic processing, we preprocessed the Wi-Fi data by removing such duplicate scan records to ensure that each data point used for localization reflects the current network environment. The main configuration parameters of the fingerprint database and the test trajectory are summarized in Table 3.

To obtain a reliable ground-truth (GT) trajectory for quantitative evaluation, we established a local 2-D Cartesian coordinate frame aligned with the 1 m × 1 m floor grid. The origin was placed at the lower-left corner of the test area, and the tile edges defined the x- and y-axes. The coordinates of the selected Wi-Fi reference points and the vertices of the double-L test path were measured using a Leica DISTO D2 (Leica Geosystems AG, Heerbrugg, Switzerland) handheld laser rangefinder (0.05–100 m range, ±1.5 mm nominal accuracy). Each distance was measured three times and averaged, and the standard deviation of the repeated measurements was below 5 mm, which is negligible compared with meter-level localization errors. These surveyed vertices were then connected along the corridor centerline to form the GT trajectory as a polyline. This geometric ground truth depends only on tape-measure distances between floor tiles and is completely independent of any Wi-Fi measurements. The geometric length of this path is approximately 37 m, obtained from the number of 1 m floor-tile intervals along the double-L loop rather than from any PDR output. During the experiments, the subject walked exactly one lap along this path, resulting in 73 steps. We then placed the 73 GT step positions approximately uniformly along the GT polyline according to their step index and took the corresponding points on the polyline as the ground-truth (x, y) coordinates of each step.

With the experimental data and ground-truth trajectories prepared as described above, we now present the evaluation framework and performance metrics used to assess the proposed algorithm.

### 4.3. Evaluation Framework and Performance Metrics

To comprehensively evaluate the proposed method, we adopt a multi-level evaluation framework. At the module level, the underlying PDR component is assessed separately using step-detection accuracy. At the system level, we compare four complete localization pipelines—Wi-Fi fingerprinting, PDR, EKF-based fusion, and the proposed GA-based fusion. For these methods we report numerical metrics including mean error, RMSE, standard deviation, maximum error, median, and CEP50/CEP90/CEP95, which are summarized in tables. We also compute the Dynamic Time Warping (DTW) distance to quantify trajectory-shape similarity and the Hausdorff distance to measure worst-case deviation. Section 5.3.6 further investigates the optimization layer by comparing different optimizers (LM, GA, PSO and Procrustes alignment) and analyzing their sensitivity to initialization and robustness to Wi-Fi outliers in terms of RMSE, CEP95 and runtime. In addition, we conduct a geometry-based synthetic trajectory-alignment experiment to further isolate the behavior of GA and least-squares estimators under controllable outlier ratios, without relying on any particular fingerprint-database implementation; the details of this experiment are presented in Section 5.3.6. All offline preprocessing, algorithm implementation, and evaluation were performed using MATLAB R2021b (The MathWorks, Inc., Natick, MA, USA).

## 5. Experimental Results and Discussion

### 5.1. Evaluation of the Step Detection Algorithm

**Figure 6:**
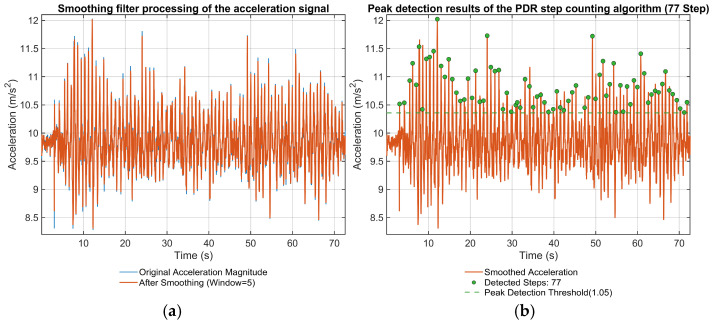
Accelerometer data processing and step detection results. (**a**) Comparison of the raw accelerometer data (blue line) and the data after applying a smoothing window filter (orange line). (**b**) The step count detected by the algorithm versus the ground truth.

To evaluate the quality of the data collected from the inertial sensors, we applied a windowed filter to the accelerometer signal. As shown in Figure 6a, the blue curve represents the raw accelerometer data, while the red curve represents the filtered data. The blue curve exhibits numerous spikes in the 8.3–12.2 m/s^2^ range. After smoothing via the filter, these spike features are reduced, while the corresponding gait features are preserved. Figure 6b shows that the step detection algorithm detected a total of 77 steps, which completely encompasses the 73 ground-truth steps. However, four distinct peaks, corresponding to 4 mis-detected steps, were observed in the 10–30 s and 45–65 s intervals, resulting in a step detection accuracy of 94.8%. Overall, the windowed filtering and step detection method achieved a high accuracy rate and a low misdetection rate, providing reliable input data for the subsequent PDR trajectory estimation and fusion localization algorithm.

For the walking experiment considered in this paper, the subject completed the precisely measured 37 m double-L path in approximately 70 s with 73 true steps. This corresponds to a mean walking speed of about 0.53 m/s, a mean step length of 37/73 ≈ 0.51 m, and a mean step rate of roughly 1.0 step/s. These values are consistent with a deliberately slow and careful gait in a narrow office corridor and form the basis for calibrating the step-length coefficient C in (9). This moderate walking speed was chosen deliberately to obtain clear gait segmentation and stable sensor signals in this first validation experiment.

We also compared the calibrated mean step length with a more general stride-length model of the form $S=KH^{a}R^{b}A_{p}^{c}$, where H is the subject height, R is the step rate (steps/s), $A_{p}$ is the peak-to-peak vertical acceleration, and the parameters are K=0.24, a=1.02, b=−0.107, and c=0.33. For our subject with H=1.75 m, R≈1.0 step/s, and observed peak-to-peak accelerations $A_{p}\in [1.5,3.0]$ m/s^2^, this model yields step-length values in the range S ≈ 0.49–0.61 m, which agrees well with the calibrated mean step length of 0.51 m. This consistency indicates that the AN-602-based model in (9), after calibration of C, provides a reasonable step-length estimate for the gait and walking speed considered in this study.

**Figure 7:**
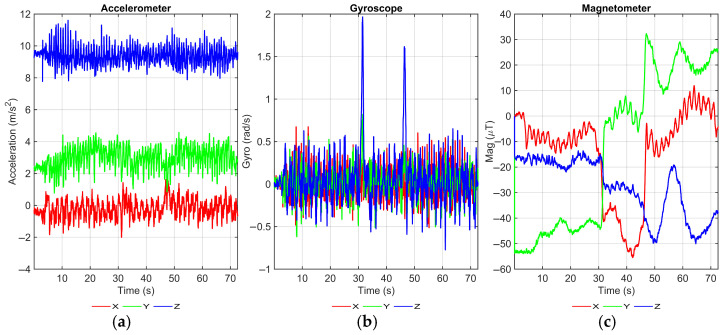
Time series of tri-axial inertial and magnetic signals during the walking trial: (**a**) accelerometer; (**b**) gyroscope; (**c**) magnetometer.

Figure 7 first presents the raw tri-axial accelerometer, gyroscope, and magnetometer signals recorded along the test path. The accelerometer data show clear periodic oscillations associated with footfalls, and the gyroscope traces exhibit two distinct peaks at approximately 30 s and 50 s, which correspond to the two turning instants in the corridor. In contrast, the magnetometer channels display pronounced nonlinear variations, especially in the interval between 45 s and 70 s, when the subject walks close to computer equipment and metallic office furniture. These variations indicate local indoor magnetic anomalies that violate the homogeneous-field assumption commonly adopted by AHRS filters and are expected to perturb magnetic-heading estimates in that part of the trajectory.

**Figure 8:**
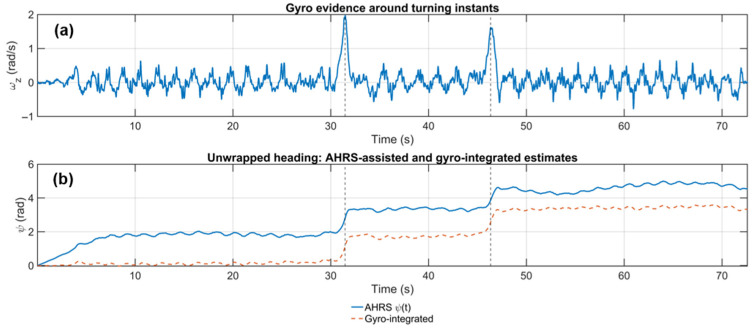
Turning detection and heading estimation: (**a**) gyroscope z-axis angular rate $\omega_{z}(t)$ around the turning instants in the corridor; (**b**) unwrapped yaw angle estimated by the Madgwick AHRS filter (intermediate PDR input) and by direct gyro integration.

Figure 8 focuses on the resulting yaw estimation. The top subplot shows the z-axis angular rate $\omega_{z}(t)$, where the two sharp spikes again mark the turning instants. The bottom subplot compares the unwrapped heading obtained from the Madgwick AHRS filter (blue line) with the heading obtained by direct gyro integration (orange dashed line). The gyro-integrated curve appears almost straight over the 45–70 s segment, which is consistent with the nearly zero angular-rate measurements during this straight-line walk; however, such pure integration is well known to suffer from cumulative drift over longer durations, even though this drift is not yet pronounced within the short 70 s interval of our trial. The AHRS filter, in contrast, fuses magnetometer observations to constrain this long-term drift. As correctly reflected in the data, this comes at the cost of local fluctuations in the 45–70 s interval: the slight waviness of the blue curve matches the magnetic anomalies observed in Figure 7 and illustrates the influence of indoor magnetic disturbances on the heading estimate.

It is important to emphasize that the AHRS output shown in Figure 8b serves only as a preliminary orientation input for the PDR module. The proposed GA-based fusion (Section 3.2) is subsequently applied at the trajectory level: it does not modify each step-wise heading estimate, but computes a global similarity transform that anchors the PDR trajectory to the Wi-Fi fingerprint coordinates, thereby compensating for both local sensor imperfections (including magnetic disturbances) and global misalignment.

### 5.2. Comparative Performance Analysis

**Figure 9:**
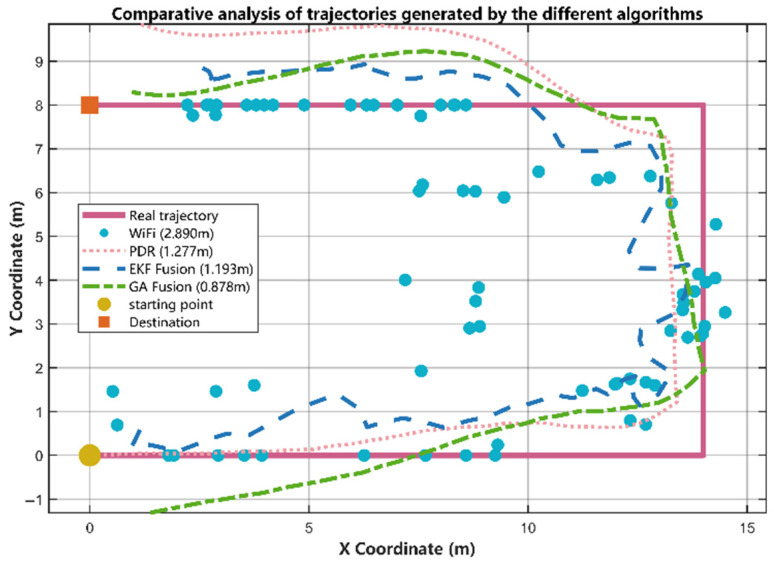
Trajectory Comparison of Different Localization Algorithms.

Figure 9 shows the trajectory comparison for the different algorithms. The red solid line represents the ground-truth trajectory obtained from precise measurements and serves as the evaluation benchmark. The results from Wi-Fi fingerprinting are represented by blue scattered points, with a mean error of 2.890 m. As seen in the plot, although the Wi-Fi positioning results are generally distributed around the true path, they exhibit strong spatial randomness and discreteness due to the influence of multipath effects, signal fading, and environmental changes. Therefore, while Wi-Fi fingerprinting can provide a global coordinate estimate, it cannot form a continuous and smooth trajectory.

The trajectory from the PDR algorithm is depicted by the pink dotted line, with a mean error of 1.277 m. It is observable that the PDR algorithm exhibits good positioning accuracy in the initial stages, with a smooth trajectory and clear local features. However, due to the accumulation of errors from the inertial sensors over time, the trajectory suffers from significant overall drift.

The EKF algorithm, a classic fusion method for Wi-Fi and PDR data, is shown as a blue dashed line, with a mean error of 1.193 m. It generates a smoother and more continuous trajectory than the two aforementioned methods. However, as a recursive linear estimator, its performance depends on the precision of both predicted and observed values. When observation accuracy is low, it still suffers from considerable errors in the later stages of positioning.

The global fusion method proposed in this paper, which uses a GA, is represented by the green dashed line. Among all compared algorithms, the GA-based fusion method performs the best. Through global optimization, it fuses the global coordinates provided by Wi-Fi fingerprinting with the clear local features from the PDR algorithm. By finding an optimal rotation and translation, it overcomes the overall drift problem inherent in PDR and provides a more accurate fused trajectory. Overall, this trajectory highly coincides with the ground truth, achieving precise tracking at the corners, with a mean error of 0.878 m. This strongly validates the core idea proposed in this paper: that fusing Wi-Fi’s global information with PDR’s precise local information can effectively generate more accurate localization results.

**Figure 10:**
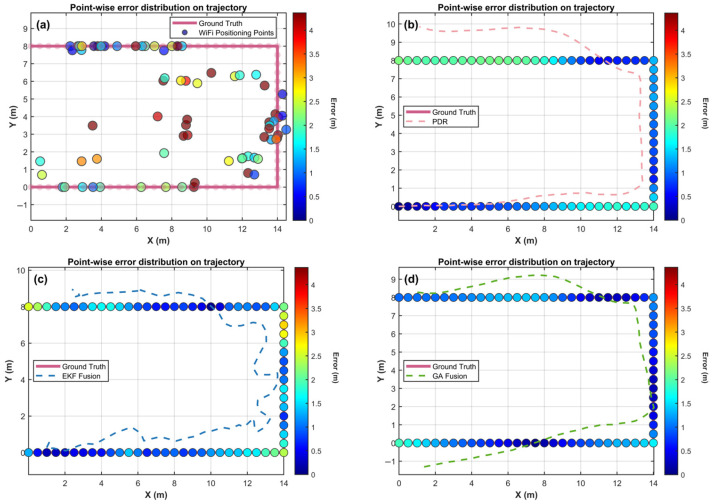
Point-wise Error Distribution of Different Positioning Methods on Trajectory: (**a**) Wi-Fi fingerprinting; (**b**) PDR; (**c**) EKF Fusion; (**d**) GA Fusion.

To better illustrate the relationship between the fused trajectory coordinates and the ground-truth coordinates, Figure 10 systematically compares the performance of four positioning methods along the actual trajectory. The pink solid line represents the ground truth, and the four subplots respectively display the positioning results of (a) Wi-Fi fingerprinting, (b) PDR, (c) EKF fusion, and (d) GA fusion. Color coding is used to represent the deviation of each estimated point from the ground truth, with error magnitudes ranging from blue (<0.5 m) to red (>4 m) across the four algorithms.

Subplot (a) shows that Wi-Fi positioning suffers from severe spatial instability, with individual points displaying highly scattered distributions. Owing to RSSI fluctuations caused by indoor multipath effects, large yellow-to-red regions indicate deviations of 2–4 m between estimated points and the true trajectory, particularly at corner and central segments of the trajectory, where peak errors exceed 4 m.

Subplot (b) illustrates that the PDR algorithm achieves good trajectory continuity owing to the high sampling rate of inertial sensors. Most positioning points remain within the blue-to-light-green range, corresponding to errors under 1.5 m, and the geometric trajectory shape generally aligns with the ground truth. In the right boundary region (x = 14–15 m), cumulative effects of gyroscope bias, accelerometer calibration errors, and stride-length estimation drift result in a noticeable color gradient, indicating a systematic increase in positioning error.

Subplot (c) shows that the EKF algorithm, leveraging state prediction and observation update mechanisms, achieves sub-meter accuracy for approximately 85–90% of the trajectory. However, in the lower-left region (x = 0–2 m, y = 0–2 m) and near the upper-right corner, the estimated trajectory deviates significantly. The color shifts to cyan–green, corresponding to 2–3 m of error, likely due to incorrect estimation caused by anomalies in Wi-Fi signals or PDR data, leading to degraded positioning performance.

Subplot (d) demonstrates the advantage of the proposed GA fusion algorithm, where nearly the entire trajectory lies within the blue region, corresponding to errors below 1 m. Only a few points show minor cyan transitions, and the estimated trajectory remains close to the ground truth without the local error peaks observed in the EKF results. This indicates that the global search capability and adaptability of the GA to nonlinear and non-Gaussian observations effectively suppress the influence of outlier measurements through population evolution.

From the 2.5–3.5 m errors of Wi-Fi positioning to the sub-1.5 m relative accuracy of PDR, followed by EKF’s localized optimization and GA’s global stability, the results demonstrate that achieving high-precision positioning in complex indoor environments cannot rely solely on linear filtering frameworks. Instead, intelligent optimization strategies capable of handling strong nonlinearity and resisting observation anomalies are required. This study provides a new perspective for the architectural design of indoor positioning systems.

**Figure 11:**
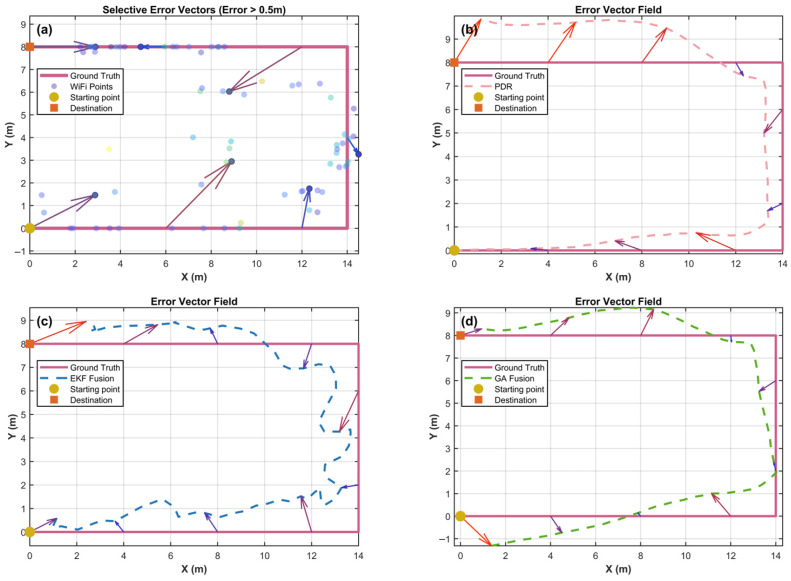
Error Vector Field of Different Positioning Methods: (**a**) Wi-Fi fingerprinting (error >0.5 m); (**b**) PDR; (**c**) EKF Fusion; (**d**) GA Fusion.

Figure 11 extends the analysis of the relationship between algorithm-generated coordinates and ground-truth coordinates by visualizing the error vector field. Compared with color maps that only represent error magnitudes, vector visualization enables further analysis of error directionality and spatial distribution patterns. Each arrow originates from the estimated position and points toward the true position; its length and direction represent the magnitude and systematic bias characteristics of the error, respectively. The four subplots respectively illustrate (a) Wi-Fi error vectors greater than 0.5 m, (b) PDR error vectors, (c) the EKF fusion error vector field, and (d) the GA fusion error field, with the pink solid line indicating the ground-truth trajectory.

In subplot (a), because of the large number of fingerprinting points, only positioning points with errors greater than 0.5 m are visualized. Light-blue scatter points are densely distributed around the ground-truth trajectory, while darker error vectors exhibit pronounced randomness. The vector lengths range from short arrows representing errors <1 m to long arrows corresponding to errors of 3–4 m. The directions are randomly distributed without spatial consistency, with higher error density observed at corners and boundary regions. The clusters of long vectors at the lower-left starting point and along the right boundary indicate fingerprint mismatches in these regions, whereas in the central region, despite dense positioning points, the irregular orientations of vectors reflect the non-stationarity of RSSI signals caused by environmental multipath effects.

Subplot (b) depicts the evolution of cumulative errors in PDR. In the initial segment (x < 6 m), almost no visible vectors appear, indicating excellent short-term accuracy. Sparse short vectors begin to appear in the middle segment, while vector lengths increase along the right and lower boundaries, exhibiting a consistent directional pattern. Specifically, vectors along the upper boundary point downward, those along the right boundary point toward the lower left, and those along the lower boundary point upward. Such directional patterns may result from gyroscope bias, which induces linear accumulation of heading-angle errors, causing the estimated trajectory to rotate relative to the ground truth. Additionally, accelerometer bias further contributes to scale distortion in displacement estimation. The longest vectors, approximately 1.5 m at the endpoint, correspond to the color gradient observed in Figure 8b, further illustrating the cumulative error characteristics of IMU sensors.

In subplot (c), the spatial distribution of error vectors exhibits distinct regional characteristics. In the mid-left boundary (x < 3 m) and upper boundary (x = 4–10 m) regions, the vectors are short and directionally scattered. Nevertheless, the EKF effectively integrates absolute Wi-Fi positioning with relative PDR constraints. The long vectors near the lower-left starting point may result from an oversized initial covariance matrix, leading to excessive reliance on the first Wi-Fi observation. The increased number of inward-pointing vectors near the upper-right corner (x ≈ 14 m, y ≈ 8 m) likely arises from abrupt Wi-Fi signal fluctuations and delayed PDR heading estimation.

Subplot (d) demonstrates the advantages of the global optimization strategy: the GA fusion algorithm’s trajectory almost completely overlaps with the ground truth, with error vector lengths remaining below 1 m throughout. The number of error vectors is also fewer than in the other methods, and their directions show no systematic orientation. When an individual solution becomes trapped in a local optimum due to Wi-Fi anomalies, the GA enables other individuals in the population to continue exploring the correct regions, thereby converging to the global optimum through fitness-driven evolution.

Table 4 presents a comprehensive performance comparison of the four localization algorithms evaluated in the experiment. The GA-based fusion algorithm proposed in this paper achieves significant improvements across all evaluation metrics. Specifically, the GA-based fusion algorithm attains a mean error of 0.878 m, representing a 69.6% improvement over Wi-Fi fingerprinting. It also shows improvements of 31.2% and 26.4% compared to the 1.277 m of the PDR algorithm and the 1.193 m of the traditional EKF fusion algorithm, respectively. Furthermore, the standard deviation and maximum error of the GA-based fusion algorithm are 0.435 m and 1.904 m, respectively, both of which are lower than those of the other methods. This demonstrates the excellent positioning consistency and stability of the proposed GA algorithm.

### 5.3. Detailed Analysis of Positioning Errors

#### 5.3.1. Statistical Analysis of Error Distribution

**Figure 12:**
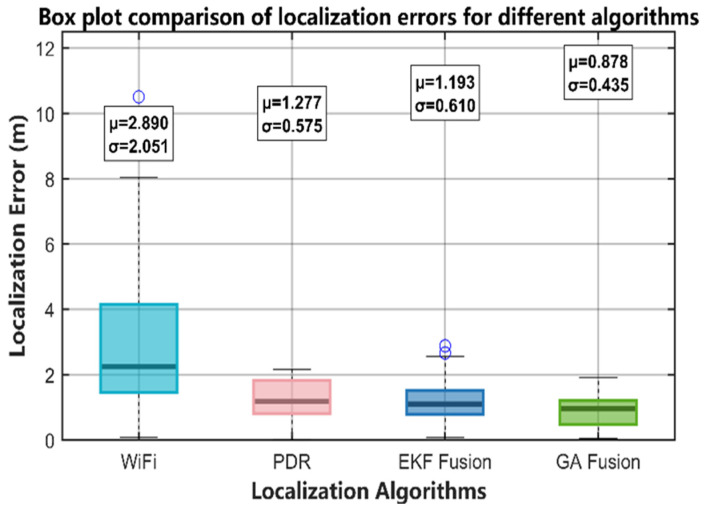
Box Plots of Localization Errors for Different Algorithms.

To further quantitatively compare the error-distribution characteristics of each algorithm, Figure 12 presents a box plot of their localization errors, clearly illustrating performance differences in terms of error concentration, fluctuation range, and outlier suppression. The error distribution for Wi-Fi fingerprinting is the most dispersed, indicated by the widest box and the presence of an extreme outlier. This confirms that RSSI measurements are susceptible to multipath effects, which can cause sudden, large errors. In comparison, the PDR algorithm’s error distribution is more concentrated, represented by a narrower box; however, the longer upper whisker reveals the inherent problem of cumulative drift errors. The traditional EKF fusion algorithm further reduces the size of the error box, showing more stable performance. Nevertheless, the several remaining outliers indicate that the EKF has limited capability in handling the non-Gaussian observation noise from Wi-Fi.

In contrast, the GA-based fusion algorithm proposed in this paper exhibits the optimal performance. Its box is the most compact, its median line is the lowest, and it has no outliers, demonstrating the method’s exceptional robustness and stability. Overall, the error-distribution patterns shown in Figure 9 are highly consistent with the quantitative metrics in Table 3. From a statistical distribution perspective, this again proves that the proposed GA-based global optimization algorithm outperforms traditional algorithms in terms of localization accuracy, outlier suppression, and system stability.

#### 5.3.2. Evaluation of Continuous Tracking Stability

**Figure 13:**
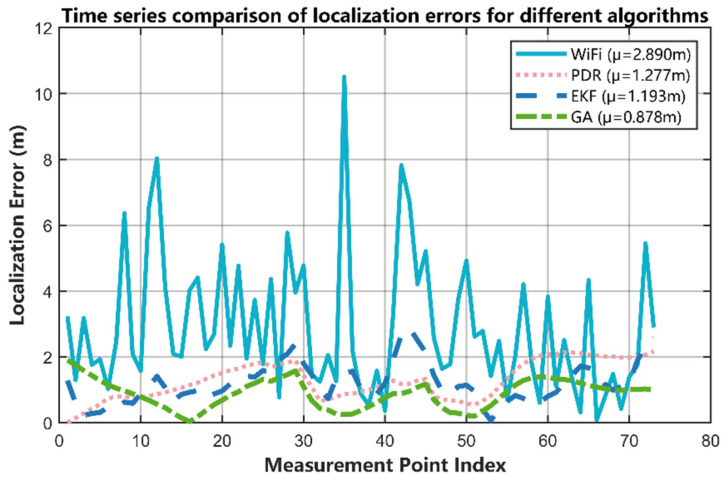
Time-series of Localization Errors for Different Algorithms.

Figure 13 presents a comparison of the instantaneous time-series errors of the different algorithms as a function of the measurement steps. The blue solid line represents the error time series for Wi-Fi fingerprinting, which exhibits high-frequency, large-amplitude fluctuations and remains at a very high level throughout the experiment. This is consistent with the characteristic of Wi-Fi fingerprinting being highly susceptible to environmental factors. The pink dashed line represents the error time series for the PDR algorithm. Overall, it shows fewer fluctuations and the curve is smoother; however, significant jumps are observed toward the end of the experiment, which aligns with the PDR algorithm’s tendency to accumulate sensor errors that affect trajectory estimation.

The blue dashed line represents the time-series error for the EKF algorithm. Although the EKF can effectively suppress the influence of uncertain environmental factors, abrupt error spikes are still observed during the process, indicating that the algorithm experiences deviations during its state estimation. Notably, the time-series error curve for the GA-based fusion algorithm proposed in this paper is represented by the green dashed line. It remains at the lowest level throughout the entire experiment, exhibiting only minor fluctuations and demonstrating excellent robustness and stability. This figure indicates that the proposed fusion algorithm not only achieves a significant improvement in overall localization accuracy but is also capable of providing continuous, high-precision trajectory tracking.

#### 5.3.3. Analysis of Error Convergence and Segment Wise Performance

**Figure 14:**
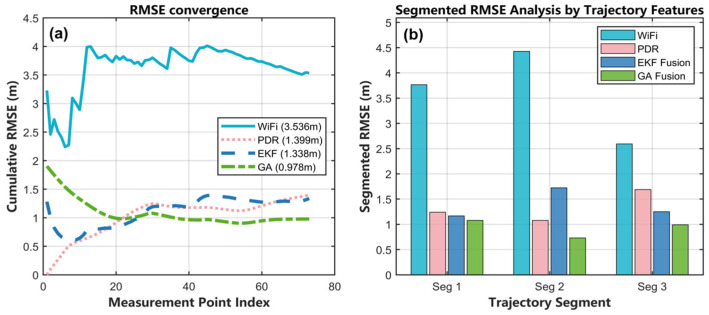
Analysis of Error Convergence and Segment wise Performance. (**a**) Cumulative RMSE convergence of different localization methods along the trajectory; (**b**) Segment-wise RMSE comparison of different localization methods for three trajectory segments.

Figure 14a illustrates the cumulative RMSE convergence characteristics of the four positioning algorithms as a function of the measurement-point index. The GA-based fusion method achieved the lowest RMSE of 0.978 m, demonstrating superior convergence performance compared with EKF fusion (1.340 m), the PDR algorithm (1.399 m), and Wi-Fi fingerprinting (3.536 m). After approximately the first 10 measurement points, the RMSE fluctuations become minimal. This rapid stabilization arises from its ability to process the entire trajectory segment simultaneously, rather than updating estimates sequentially point by point.

In contrast, the Wi-Fi fingerprinting method exhibits severe instability, as the RMSE fluctuates dramatically between 2.2 m and 4 m throughout the measurement sequence owing to multipath propagation and environmental dynamics affecting signal strength. The PDR method starts with near-zero error but shows a clear upward error trend, eventually reaching an RMSE of 1.399 m and causing the estimated trajectory to gradually drift from the ground truth—demonstrating the inherent error-accumulation problem of inertial navigation systems without external corrections. The EKF fusion method converges to 1.340 m after an initial transition period of 30–40 measurement points. The recursive Bayesian estimation effectively mitigates Wi-Fi measurement noise but remains sensitive to initialization and measurement outliers.

Figure 14b analyzes the trajectory by dividing it into three segments, where the GA fusion method maintains the lowest RMSE across all segments, as detailed in Table 5. This demonstrates its robustness to different trajectory characteristics such as turning and straight walking. The errors of the PDR and EKF methods show a gradual increase from segment 1 to segment 3, reflecting their typical cumulative-error behavior. The Wi-Fi method maintains consistently high and relatively uniform errors, reflecting its globally available but low-accuracy nature.

The convergence and segmentation analyses collectively indicate that the proposed GA-based fusion algorithm effectively overcomes the limitations of traditional filtering methods. It achieves rapid convergence without dependence on initial states, delivers superior overall accuracy, and provides more stable and reliable tracking performance across the entire trajectory.

#### 5.3.4. Trajectory Geometric Quality Assessment

**Figure 15:**
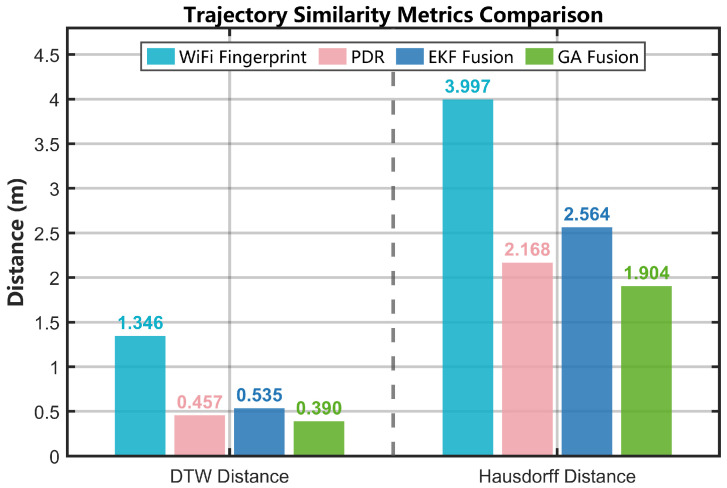
Trajectory similarity metrics comparison using DTW and Hausdorff distances.

Figure 15 illustrates the convergence behavior and segmented performance of the different algorithms, focusing primarily on pointwise positioning errors. To comprehensively assess the quality of the trajectories, two geometric similarity metrics were employed: the DTW distance for evaluating shape fidelity and the Hausdorff distance for assessing worst-case deviation.

Due to scanning latency, Wi-Fi RSSI measurements are typically obtained at a frequency of 1 Hz, whereas IMU data are continuously streamed at 50–100 Hz. DTW aligns these asynchronous data streams by optimizing point-to-point correspondence based on geometric similarity rather than temporal proximity, enabling comparison across datasets with differing sampling densities.

The GA fusion algorithm achieved the lowest DTW distance of 0.390 m, improving by 71.0% over Wi-Fi fingerprinting (1.346 m), 14.7% over PDR (0.457 m), and 27.8% over EKF fusion (0.540 m). The remarkably low DTW distance achieved by the GA method indicates that the global optimization successfully preserves the intrinsic geometric structure of the PDR trajectory while partially correcting cumulative drift.

The Hausdorff distance, a key metric for measuring maximum trajectory deviation, shows that the proposed GA fusion method limits the maximum deviation to 1.904 m, outperforming Wi-Fi fingerprinting (3.997 m), PDR (2.168 m), and EKF fusion (2.564 m). In particular, the 52.4% reduction in maximum deviation compared with Wi-Fi positioning strongly demonstrates that the algorithm effectively suppresses the influence of extreme outliers through the application of the Huber loss function.

Notably, although EKF achieves higher average accuracy, its Hausdorff distance (2.564 m) exceeds that of PDR (2.168 m), indicating that when an erroneous Wi-Fi observation severely perturbs the state estimate, the filter produces deviations that require multiple subsequent cycles to recover. In contrast, the GA’s global batch optimization mechanism simultaneously considers all observational data, effectively preventing such error propagation.

#### 5.3.5. Reliability Analysis Using Error CDF

Single scalar metrics such as the mean error cannot fully characterize a localization algorithm’s accuracy and reliability. Therefore, we also use the cumulative distribution function (CDF) of the positioning error for a more in-depth and comprehensive evaluation. In general, the steeper a CDF curve and the closer it is to the top-left corner, the smaller the errors and the more stable the performance.

**Figure 16:**
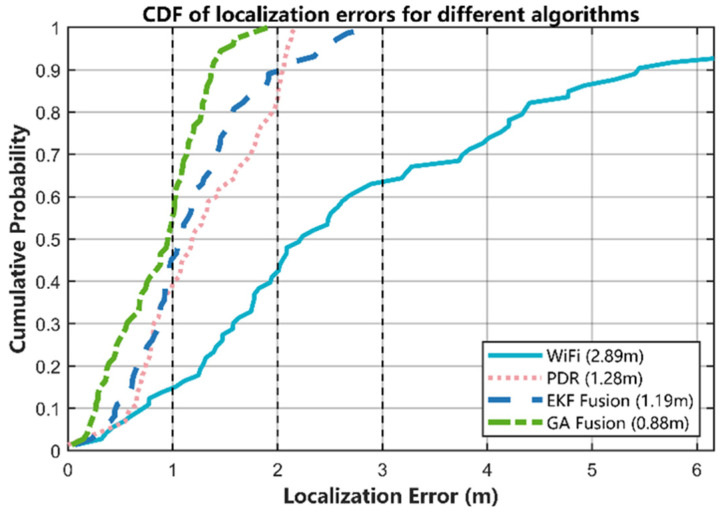
CDF of Localization Errors for Different Algorithms.

As shown in Figure 16, the CDF curves of the four methods exhibit a clear separation. The curve of the proposed GA-based fusion is the steepest and lies consistently above the others. The Wi-Fi fingerprinting curve is located in the bottom-right region, indicating the worst performance, while the EKF and PDR curves fall in between but still show a noticeable gap relative to GA. As can be seen from Table 4, at the 50th, 80th, 90th, and 95th percentiles of the error distribution, the GA-based fusion achieves the smallest errors among all methods, demonstrating both high accuracy and excellent stability. It is worth noting that, even in this office-scale environment with relatively dense AP deployment and mostly line-of-sight links along the 37 m double-L path, the Wi-Fi CDF still extends beyond 10 m of error, confirming that RSSI fingerprinting remains a noisy global position source.

From another perspective, if we set a specific error tolerance—for instance, a target accuracy of 1.5 m—Table 6 and the CDF plot together show that the GA-based fusion satisfies this requirement with about 90% confidence. In contrast, EKF and PDR reach this accuracy level for only around 50% of the samples, and the Wi-Fi fingerprinting method remains below 50%. This indicates that, even under the same error tolerance, the proposed GA-based fusion consistently provides superior localization accuracy, reliability, and consistency compared with the other three methods.

#### 5.3.6. Sensitivity and Robustness Analysis of Alignment Optimizers

**Figure 17:**
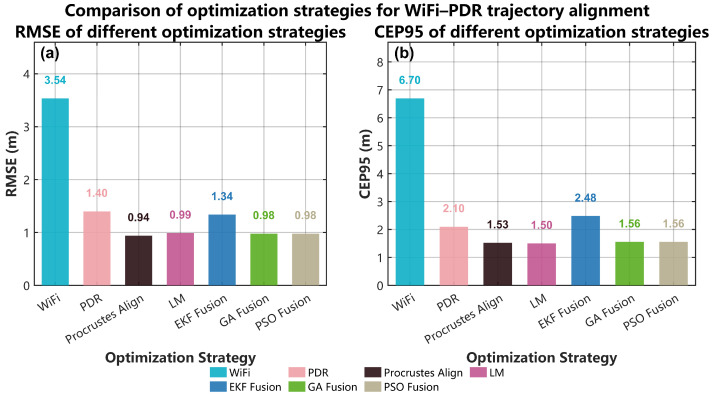
Comparison of optimization strategies for Wi-Fi/PDR trajectory alignment: (**a**) RMSE and (**b**) CEP95 of different optimization strategies.

**Figure 18:**
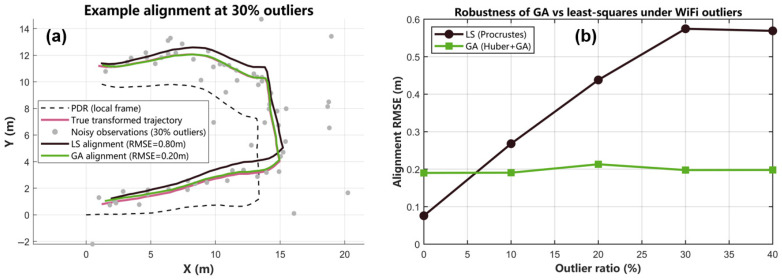
Geometry-based synthetic trajectory-alignment experiment using a real PDR path: (**a**) Example alignment result with 30% outlier-contaminated absolute position observations. (**b**) Alignment RMSE of least-squares Procrustes and GA estimators versus outlier ratio.

This subsection analyzes the behavior of different trajectory–alignment optimizers from both a system-level and an algorithmic perspective. First, using the real Wi-Fi/PDR data collected in our office environment, we compare Procrustes alignment, LM, the proposed GA-based fusion, and PSO-based fusion in terms of positioning accuracy, sensitivity to initialization, runtime and robustness to Wi-Fi outliers (Figure 17 and Table 7 and Table 8). Then, to isolate the effect of the optimizer itself from the particular Wi-Fi fingerprint implementation, we design an additional synthetic trajectory-alignment experiment based on a real PDR path but with controlled outliers in the absolute position observations (Figure 18).

Figure 17a,b summarizes the localization accuracy of different optimization strategies in terms of RMSE and CEP95, respectively. In addition to the baselines of Wi-Fi fingerprinting, PDR, and EKF fusion, four trajectory–alignment methods are evaluated: Procrustes alignment, LM, the proposed GA-based fusion, and PSO-based fusion. Procrustes alignment directly uses the ground-truth trajectory as the reference, and therefore can be regarded as an upper bound under ideal geometric alignment. For the other three optimizers, all methods achieve sub-meter accuracy on this four-parameter similarity–transform problem: the RMSE lies in the range of 0.94–0.99 m and CEP95 is around 1.50–1.56 m. This indicates that, in our complex office scenario, the objective landscape is relatively benign, and the GA and PSO methods do not lose localization accuracy compared with the classical LM solver.

Table 7 further investigates the sensitivity of each algorithm to initialization and its runtime. LM is tested with a good initial guess and with a deliberately perturbed bad initial guess. In both cases LM converges to almost the same solution, which suggests that, under the current cost function, the problem is well conditioned and LM is unlikely to be trapped in poor local minima. In contrast, GA and PSO do not rely on any prior initial state or Jacobian. They start from randomly generated populations or particle swarms, and still converge to an accuracy level comparable to that of LM. The main difference lies in computational time: in our implementation and hardware platform, LM takes about 0.01 s per run, PSO about 0.17 s, whereas GA requires roughly 3.23 s per run because of its larger population size and number of generations. Therefore, we treat the GA-based fusion as an offline trajectory post-processing step rather than a real-time filter, and we point out that hybrid schemes combining GA with fast local refinement are an important direction for future work.

Table 8 evaluates the robustness of the optimizers to Wi-Fi outliers. We inject 30% artificial outliers into the Wi-Fi trajectory and rerun the alignment. As expected, the Procrustes solution is almost unaffected, because it aligns the PDR trajectory directly to the ground-truth path instead of to noisy Wi-Fi positions; it should therefore be interpreted as an ideal upper bound rather than a deployable algorithm. Among the methods that rely on Wi-Fi measurements, LM shows the largest degradation in the presence of outliers: its CEP95 increases from 1.505 m to 1.753 m. In contrast, the GA- and PSO-based fusions employ the Huber loss and exhibit much smaller changes in CEP95, from 1.558 m to 1.541 m and from 1.557 m to 1.546 m, respectively. These results demonstrate that the proposed robust cost function can effectively limit the influence of large Wi-Fi errors on trajectory alignment and improve the stability of the fused localization under challenging conditions.

Overall, the experiments in this section show that, for the current low-dimensional similarity-transform problem, the three optimization methods reach a similar accuracy level. At the same time, GA and PSO offer additional advantages: they do not depend on carefully tuned initial guesses and are easier to extend to higher-dimensional parameters and complex constraints. This justifies the use of a GA-based global optimization framework as the core module of our Wi-Fi/PDR trajectory fusion method.

In order to investigate the behavior of the trajectory–alignment optimizers without relying on a particular Wi-Fi fingerprinting implementation, we select one real PDR trajectory from the dataset and treat its 2-D local coordinates as the underlying true path. A known similarity transform (scale 1.05, rotation 8°, translation [1.2, 0.8] m) is then applied to obtain an ideal global trajectory $z_{\mathrm{clean}}$. On top of this, we add zero-mean Gaussian noise and randomly replace a certain proportion of samples with large deviations to generate synthetic absolute position observations $z_{\mathrm{noisy}}$, which emulate Wi-Fi measurements contaminated by gross errors. Each optimizer only has access to the local PDR trajectory and $z_{\mathrm{noisy}}$; its task is to estimate the similarity-transform parameters between the two trajectories, and the final alignment accuracy is evaluated with respect to $z_{\mathrm{clean}}$.

Figure 18a illustrates a representative outcome for the case of 30% outliers. The GA solution with the Huber loss almost perfectly follows the ideally transformed trajectory, whereas the least-squares Procrustes solution is clearly pulled towards the corrupted observations and exhibits a visible deviation from the true path. Figure 18b reports Monte Carlo statistics over different outlier ratios. When no outliers are present, the classical LS estimator achieves the smallest RMSE (about 0.08 m), which is consistent with its optimality under purely Gaussian noise. However, once 10–40% of the observations are contaminated, the RMSE of LS rapidly increases to about 0.57 m, while the GA-based estimator remains around 0.20 m and is almost unaffected.

This controlled experiment, whose geometric structure is derived from a real walking trajectory but is decoupled from any specific fingerprint-database noise, demonstrates that the proposed GA and Huber formulation is significantly more robust to large observation errors than the standard least-squares alignment. Together with the real-world results in Table 7 and Table 8, it provides independent algorithm-level evidence in favor of using GA as the core optimizer in our Wi-Fi/PDR trajectory fusion framework.

## 6. Conclusions and Future Work

Indoor localization is of considerable scientific and practical interest. To reduce the strong dependence of multi-sensor fusion—especially EKF-based methods—on accurate initialization, this work reformulates Wi-Fi/PDR fusion as a global trajectory alignment problem and solves it with a Genetic Algorithm (GA). The proposed method searches for the optimal 2D similarity transform that rotates, scales, and translates the locally consistent PDR trajectory to match the Wi-Fi coordinate frame, yielding a globally consistent fused track. Although RSSI-based Wi-Fi fingerprinting is used as the absolute positioning source in our experiments, the formulation is sensor-agnostic and can be directly extended to other absolute measurements such as Wi-Fi RTT, UWB, BLE, and geomagnetic fingerprints. In all these cases, the role of GA fusion is the same: to turn sparse, noisy global fixes and a drifting PDR track into a globally consistent trajectory.

The main findings can be summarized as follows:

(1) Improved geometric consistency of trajectories. Among the four localization pipelines—Wi-Fi fingerprinting, PDR, EKF fusion, and GA fusion—the GA-based trajectory shows the best geometric agreement with the ground truth, with most point-wise errors below 1 m. In contrast, Wi-Fi suffers from 3–4 m deviations due to multipath, PDR exhibits accumulative drift, and EKF can be locally unstable under poor initialization and Wi-Fi outliers. By globally optimizing the similarity transform, GA fusion preserves both the shape and the orientation of the true path more faithfully.
(2) Improvement in positioning accuracy and stability. In the office environment, GA fusion achieves a mean error of 0.878 m and an RMSE of 0.978 m, which corresponds to improvements of 69.6%, 31.3%, and 26.4% over Wi-Fi, PDR, and EKF, respectively. It also attains the lowest DTW distance (0.390 m)—improvements of 71.0%, 14.7%, and 27.8%—and the smallest Hausdorff distance (1.904 m), with a 52.4% reduction compared with Wi-Fi. At a 90% confidence level, 90% of the positioning errors lie within 1.5 m, confirming the robustness and convergence efficiency of the proposed method in a multipath-rich indoor scenario.
(3) Optimizer comparison experiments show that LM, GA, and PSO all achieve sub-meter accuracy on the current four-parameter problem under clean data, but behave differently under challenging conditions. LM is fast and relatively insensitive to reasonable initial guesses, yet its CEP95 degrades noticeably when 30% artificial Wi-Fi outliers are injected. In contrast, GA and PSO, which use a Huber-based robust cost, maintain much more stable CEP95 values. Error-vector analysis further reveals that GA fusion produces short, nearly isotropic error vectors along the entire path, whereas Wi-Fi and EKF errors tend to cluster near corners and boundaries. These results support the use of GA-based global optimization as a robust fusion core.

This study also suggests several directions for further research. First, the current GA fusion is implemented as an offline post-processing step and does not yet meet strict real-time requirements. As shown by the runtime comparison in Section 5.3.6, GA is slower than LM and PSO on the present 73-step trajectory, although it remains sufficiently fast for offline analysis. In future work, we plan to investigate hybrid schemes that combine GA with faster local optimizers or incremental updates, in order to retain robustness while reducing runtime and moving towards near real-time applications. Since the cost function is a sum over all Wi-Fi–PDR point pairs, the computational cost scales approximately linearly with trajectory length under a fixed GA configuration; evaluating this scalability on longer and more complex paths is part of our ongoing research.

Second, the current optimization only exploits Wi-Fi measurements and IMU inertial data and does not explicitly incorporate map constraints or other environmental signals. In future work, we plan to integrate physical information from floor plans (e.g., that trajectory points cannot fall inside walls and should remain within walkable areas) together with additional sensing modalities such as geomagnetic fingerprints, Bluetooth beacons, and visible-light positioning into the cost function. Within the proposed fusion method, these sources can be modeled either as soft penalty terms when a candidate trajectory violates a constraint, or as additional observation residuals. This unified treatment is expected to enable multi-source data fusion in a single optimization framework, further suppress unrealistic solutions and improve both positioning accuracy and robustness.

Finally, the experiments in this paper are conducted in a single 2D indoor office environment with one subject, one handheld device, and one double-L trajectory, and the analysis is focused on planar positions. This office-scale setup was deliberately chosen as a representative yet controlled testbed for studying the behavior of the GA-based global alignment in detail. Therefore, the present experiments should be regarded as a proof-of-concept evaluation of the proposed alignment framework rather than a comprehensive validation across all indoor scenarios. Nevertheless, a comprehensive assessment of the generality of the proposed method will require more diverse users, walking speeds, trajectories, and environments. Moreover, the PDR module currently relies on a stride-length model with a subject-specific calibration coefficient C, which is acceptable for the present single-subject study but not ideal for large-scale deployment. As an extension, we aim to integrate additional signals such as barometric pressure for floor identification and vertical displacement, generalize the trajectory alignment to 3D space for multi-floor and other complex indoor layouts, and replace the personalized stride-length calibration with more general models that explicitly depend on height, step rate, and acceleration amplitude and can be shared across users. Evaluating the proposed method on more users, motion patterns, and environments will be an important part of our ongoing and future work.

## Figures and Tables

**Table 1:** Configuration of the GA-based trajectory optimizer.

Item	Symbol/Setting	Value Used in Experiments	Remark
Optimization variables	X = [α, θ, tx, ty]T	4-dimensional similarity transform	Scale, rotation, translation
Scale factor range	α	[0.97, 1.03]	Step-length bias prior
Rotation range	θ	[−*π*/16, *π*/16]	≈±11.25° heading error
Translation range	tx, ty	[−1.5, 1.5] m	Expected Wi-Fi drift
Population size		500	Same for all datasets
Max. generations		800	Termination condition
Selection operator		Tournament selection	Preserves fitter individuals
Crossover operator		Simulated binary crossover	Produces new candidates
Mutation operator		Gaussian mutation on each gene	Maintains diversity
Elitism		Best individual copied to next generation	Avoids loss of best solution
Fitness function		Cost *J*(*X*)	Defined in Section 3.2.2

**Table 2:** Experimental System Hardware Configuration.

Category	Parameter	Specification	Configuration Value
I. Test Environment	Area	Indoor office	14 m × 9 m
	Environment Type	Complex multipath	Corridors, desks, electronics
	Grid Resolution	Supported	Supported
II. Wi-Fi Infrastructure	Number of APs	Dual-model deployment	Xiaomi AX3000T (4 units),TP-Link AX3000 (4 units)
	AP Models	Supported	Supported
	Frequency Bands	Dual-band	2.4 GHz/5 GHz
	Antenna Configuration	External antennas	4 × external, 2 × 2 MIMO
III. Data Collection Device	Device Model	Mobile tablet	HONOR ViewPad 6 (Honor Device Co., Ltd., Shenzhen, China)
	Processor	SoC	Kirin 985
	Memory	RAM + Storage	6 GB + 64 GB
	IMU Sensors	Embedded sensors	Accelerometer (3-axis),Gyroscope (3-axis),Magnetometer (3-axis)
	IMU Sampling Rate	Sensor frequency	100 Hz
	Operating System	Software platform	HarmonyOS 2.0.0 (Huawei Technologies Co., Ltd., Shenzhen, China)

**Table 3:** Experimental Data Collection and Trajectory Configuration Parameters.

Category	Parameter	Value	Description
I. Fingerprint Database	Number of RPs	60 points	Reference points along closed path
	RP Spacing	1 m	Average distance between RPs
	Collection Duration per RP	2 min	RSSI stabilization period
	RSSI Sampling Rate	1 Hz	Wi-Fi scan frequency
	Fingerprint Averaging	Mean RSSI	Average over 120 samples per AP
II. Test Trajectory	Trajectory Shape	Double-L path	Designed within fingerprint area
	Number of Steps	73 steps	Continuous walking data
	Walking Speed	1 step/s	Constant pace (~0.5 m/s)
	Trajectory Length	37 m	Total path distance
	Operating System	Software platform	HarmonyOS 2.0.0

**Table 4:** Performance Comparison of Different Localization Algorithms.

Algorithm	RMSE (m)	Mean Error (m)	Standard Deviation (m)	Maximum Error (m)	Median (m)
Wi-Fi Fingerprinting	3.536	2.890	2.051	10.509	2.239
PDR	1.399	1.277	0.575	2.168	1.187
EKF Fusion	1.340	1.193	0.610	2.881	1.100
GA Fusion	0.978	0.878	0.435	1.904	0.952

**Table 5:** Segmented RMSE Analysis by Environmental Characteristics.

Segment	Environment Characteristics	Wi-Fi (m)	PDR (m)	EKF (m)	GA (m)
Segment 1	Corridor area near conference rooms with sparse obstacles	3.765	1.239	1.172	1.078
Segment 2	Open office area with dense desks and chairs, high multipath	4.425	1.077	1.722	0.730
Segment 3	Corridor at office area periphery with moderate obstacle density	2.594	1.688	1.251	0.993
Overall	Complete test path	3.536	1.399	1.340	0.978

**Table 6:** Positioning Accuracy at Different Confidence Levels.

Algorithm	Error at 50% Confidence (m)	Error at 80% Confidence (m)	Error at 90% Confidence (m)	Error at 95% Confidence (m)
Wi-Fi Fingerprinting	2.24	4.36	5.51	6.70
PDR	1.19	1.97	2.06	2.10
EKF Fusion	1.10	1.58	2.10	2.48
GA Fusion	0.95	1.28	1.38	1.56

**Table 7:** Sensitivity of different optimizers to initialization and runtime.

Algorithm	Initialization	RMSE (m)	CEP95 (m)	Runtime (s)
LM	good	0.988	1.505	0.01
LM	bad	0.988	1.505	0.01
GA fusion (Huber)		0.978	1.558	3.23
PSO fusion (Huber)		0.978	1.557	0.17

**Table 8:** Robustness of different optimizers to Wi-Fi outliers.

Algorithm	RMSE (m)	CEP95 (m)	RMSE (m)	CEP95 (m)
	Clean Wi-Fi		30% Wi-Fi outliers	
Procrustes	0.940	1.527	0.940	1.527
LM	0.988	1.505	1.034	1.753
GA fusion (Huber)	0.978	1.558	1.058	1.541
PSO fusion (Huber)	0.978	1.557	1.060	1.546

## Data Availability

The data presented in this study are available on reasonable request from the corresponding author. The data are not publicly available because they are part of an ongoing research project and are subject to our institution’s data-privacy and security policies.
